# Identification of the Fracture Process in Gas Pipeline Steel Based on the Analysis of AE Signals

**DOI:** 10.3390/ma15072659

**Published:** 2022-04-04

**Authors:** Grzegorz Świt, Ihor Dzioba, Anna Adamczak-Bugno, Aleksandra Krampikowska

**Affiliations:** 1 Faculty of Civil Engineering and Architecture, Kielce University of Technology, 25-314 Kielce, Poland; gswit@tu.kielce.pl (G.Ś.); akramp@tu.kielce.pl (A.K.); 2 Faculty of Mechatronics and Mechanical Engineering, Kielce University of Technology, 25-314 Kielce, Poland; pkmid@tu.kielce.pl

**Keywords:** gas pipeline steel, fracture process, acoustic emission, *k-means* algorithm, time-frequency analysis, wavelet analysis

## Abstract

The paper presents the results of tests conducted to identify the damage process in specimens collected from the steel of a gas pipeline. The tests concerned specimens made of S235 steel subject to quasi-static loading—uniaxial tension until failure. Acoustic emission (AE) signals were recorded during the loading process along with force and elongation signals. Sections were collected from previously loaded specimens and subjected to microstructural examinations to determine the nature of material damage at different strain stages. The recorded AE signals were analyzed using the *k-means* clustering method, as well as time-frequency analysis. The results of metallographic tests and analysis of AE signals identified frequency spectra characteristic of different stages of the process of material damage.

## 1. Introduction

Gas pipelines are intended for long-term use—some sections have a service life of 50 years or more. During long-term operation, the metal of the pipes is continuously exposed to mechanical loads, corrosive factors and hydrogen influence [1]. Mechanical loads appear due to the internal pressure of gas and external loads, including permanent, cyclical and accidental loads [2,3]. The action of corrosion [4] and hydrogen on the pipeline metal, in turn, is caused by the gas conveyed in the pipeline [5,6,7]. The long-term flow of the above-mentioned media causes specific changes in the microstructure of the pipe steel—changes in phase composition and coagulation of precipitates, the appearance of microcracks and fractures caused by corrosion and additional pressure at the tips of local micro defects caused by the action of hydrogen [8,9,10,11]. When the above-mentioned factors occur in the steel of the pipeline, they reduce its mechanical characteristics, plasticity and fracture toughness [12,13,14,15].

The standard operating conditions of gas pipelines do not provide for their emergency failures. Unfortunately, such emergencies occur from time to time. The causes of accidental failures may be, first of all, manufacturing errors, material imperfections, changes in operating conditions resulting from a sudden increase in expected loads (e.g., at road crossings, railway crossings, in areas of mining damage). To prevent such accidents, the pipeline condition was monitored using non-destructive methods [16,17,18,19,20]. Recently, such observations are increasingly performed using methods based on the analysis of acoustic emission signals (AE) [20,21,22].

Monitoring based on the analysis of AE signals enables a high-quality assessment of the condition of pipeline metal because contemporary systems used to record and analyze AE signals can record not only the damage growth but also the processes that precede it—such as plastic strain, decohesion between inclusions and the material matrix and formation of micro defects. If these processes are identified in the metal during the monitoring activities, the risks can be detected at earlier stages of the damage process and potential failure prevented. However, to use this approach in practice, it is necessary to carry out very subtle laboratory tests to create a suitable database of AE signal parameters characteristic to the relevant stage of damage [21,22].

Every damage process occurring in the material, structural member or structure is a source of acoustic emission with characteristic parameters of the recorded signal AE. The existing conventional methods for the analysis of AE signals, based on the energy analysis or the analysis of individual parameters, e.g., amplitude, energy or duration, carry significant errors due to the approximate nature of the current theories and their implementation [23,24,25,26]. The theories and solutions presently used involve some simplifying assumptions that result in significant errors in the assessment of the propagation speed of forms of elastic waves, particularly for high-frequency signals [26,27,28,29,30,31,32,33]. When individual acoustic emission parameters are used without a specific methodology, there is a high risk of making a wrong assessment of the investigated structures and the related damage processes [33].

Consequently, it was found that a condition assessment using the acoustic emission method should be based on statistical methods of signal clustering and time-frequency analysis [30,31,32,33].

This paper presents the results of research carried out to determine the correct analysis methods and parameters of AE signals that can be used to identify the mechanisms occurring during the fracture process in S235 pipeline steel. The tests were carried out on nine specimens, with three specimens collected from each of the three different pipelines. Since the qualitative and quantitative results of the tests are very similar for all specimens, this paper only presents the results for a single representative specimen.

## 2. Materials and Methods

### 2.1. Materials

**Figure 1:**
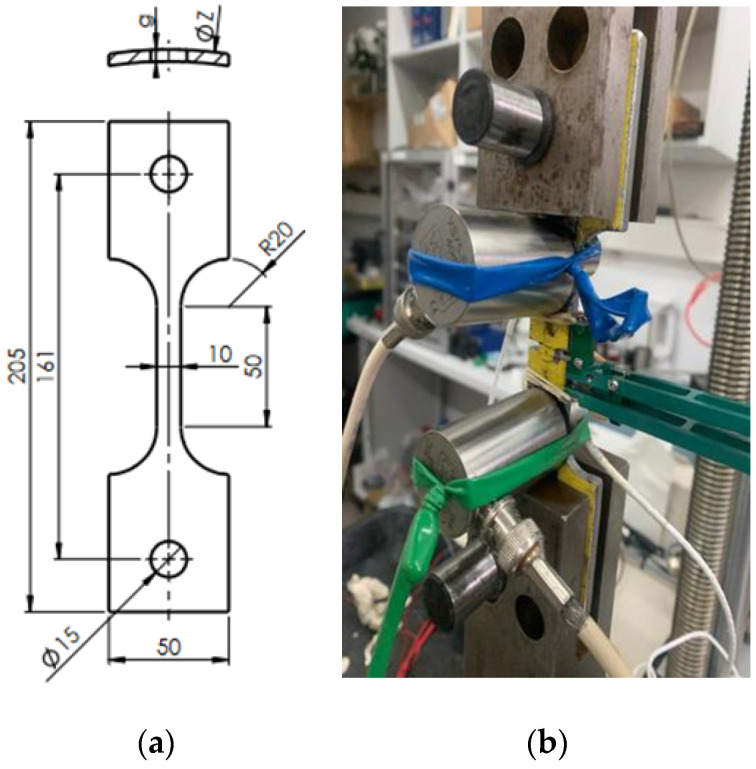
Appearance of the tested specimen: (**a**) technical drawing; (**b**) testing station with the specimen.

The tests were carried out on specimens collected directly from the sections of a pipeline intended for gas transport, a diameter of 168 mm. Due to the pipe wall thickness of about 4.0–4.5 mm, the collected specimens were flat and oriented along the pipe axis. The tested section of the specimens had the size of 10 × 50 mm (Figure 1).

**Figure 2:**
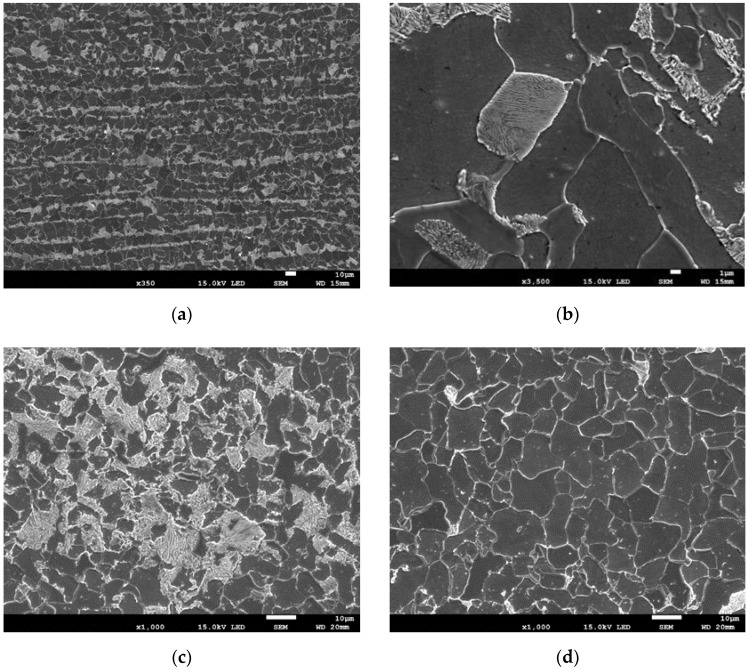
Ferritic-pearlitic microstructure of the steel in the tested pipeline: specimen across the thickness (**a**,**b**); (**c**,**d**) specimen normal to the surface (**c**) pearlite layer; (**d**) ferrite layer.

The pipeline material was S235 steel. Chemical composition of S235 steel: C ≤ 0.17; Mn ≤ 1.40; Cu ≤ 0.55; N ≤ 0.12; P ≤ 0.035; S ≤ 0.035. The metallographic tests indicated that the steel had a ferritic-pearlitic microstructure with distinct banding across the pipe wall thickness (Figure 2).

**Figure 3:**
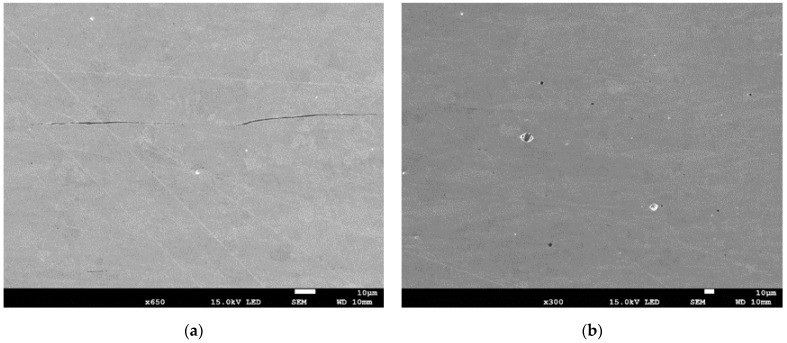
(**a**,**b**) Inclusions in the pipe material.

The images shown in Figure 2 indicate that ferrite and pearlite grains with a size of 5–15 µm are arranged in layers across the pipe wall thickness. The pipe material has inclusions of MnS in the form of thin elongated plates and spherical oxide particles with diameters of up to 10 µm (Figure 3).

### 2.2. Methods

#### 2.2.1. Specification of Material Test

The uniaxial tensile strength tests were carried out according to ASTM Standards [20] using the electrodynamic Zwick-100 (Wroclaw, Poland) machine in laboratory conditions (T = 20 ± 10C). The signals of force F (N) and test section elongation u_ext_ (mm) were recorded during the tests and used to calculate the nominal stresses, strains and plasticity characteristics—relative elongation and aria reduction.

#### 2.2.2. Acoustic Emission Method

During the uniaxial tensile strength tests, acoustic emission (AE) signals were also recorded. The AE signals were recorded using piezoelectric sensors and Express-8 system (Mistras, Physical Acoustics) [16].

During the initial stage of the tests, the sensors recorded data in four measuring ranges:

- 1–20 kHz (VS12-E);
- 30–120 kHz (VS75-SIC-40dB);
- 100–450 kHz (VS150-RIC);
- 100–900 kHz (VS900-RIC).

**Figure 4:**
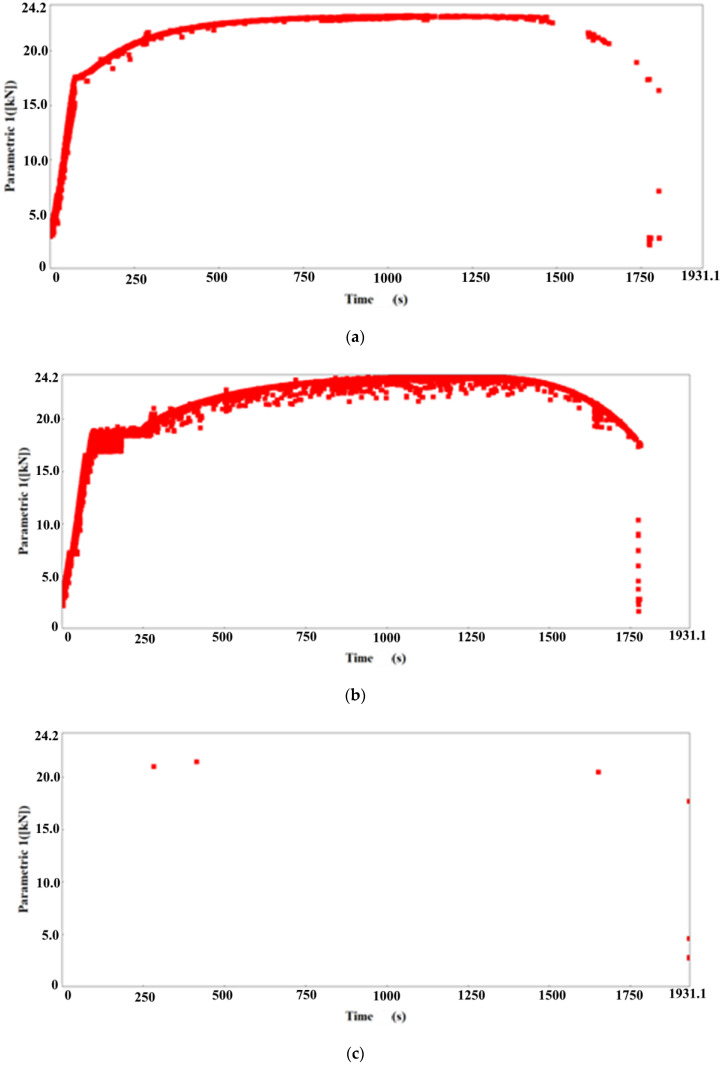

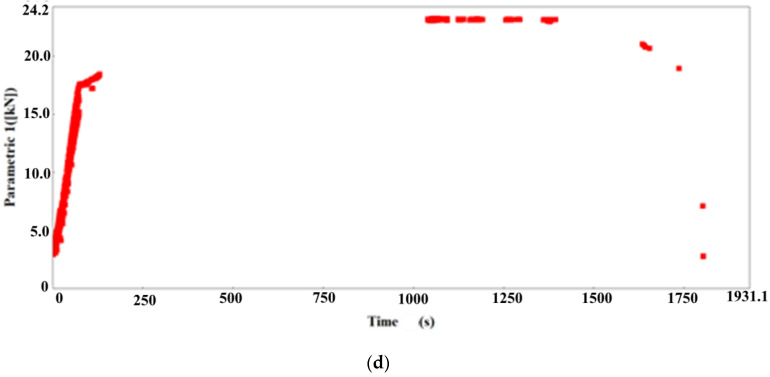
Signals recorded for the individual sensor types: (**a**) VS12-E; (**b**) VS75-SIC-40dB sensor; (**c**) VS150-RIC sensor; (**d**) VS900-RIC sensor.

Due to the frequencies recorded during the investigation of AE events, only the first two of the former sensor types were used in further tests—VS12-E and VS75-SIC-40dB. The force signals recorded by the individual sensors are shown in Figure 4.

## 3. Results and Discussion

### 3.1. Mechanical Results

**Figure 5:**
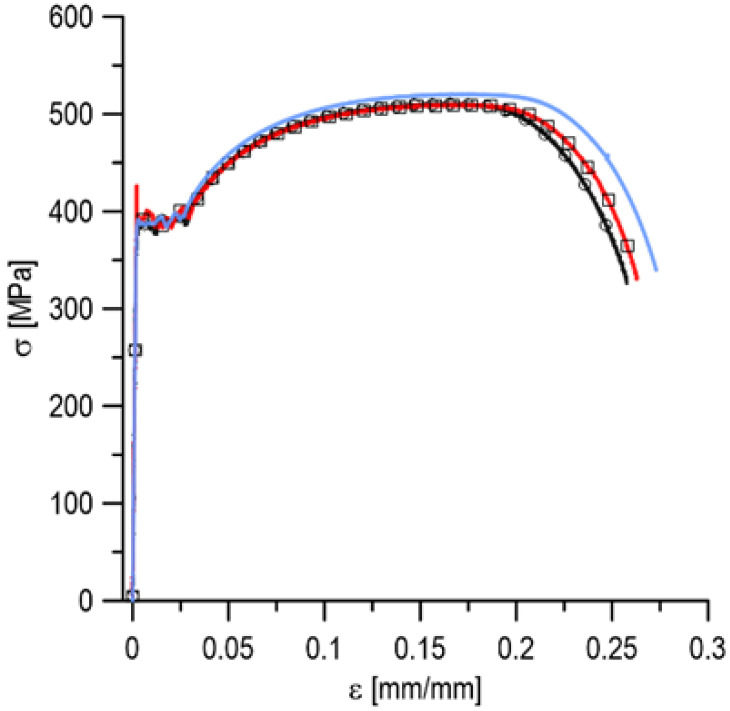
Stress–strain curves for S235 steel (different colors mean three different samples from one series).

Figure 5 shows the three stress–strain charts for the tested material in nominal values. The charts show a good agreement of the experimental results. The respective characteristic values determined based on the discussed curves are included in Table 1.

### 3.2. Description of the Fracture Process Based on Microstructural Examinations

To determine the changes occurring in the microstructure during uniaxial elongation, sections longitudinal to the plane of symmetry, located axially, were collected from the tested specimens. The collected sections were etched with a 3% HNO3 solution, and then their microstructure was observed in different parts of the elongated specimen using SEM. The purpose of the examinations was to determine the correlations between the strain of the material and the extent of damage stage: the presence of delaminations between the inclusions and the matrix; inclusion fracture; growth of voids and tearing in strained ferrite grains.

**Figure 6:**
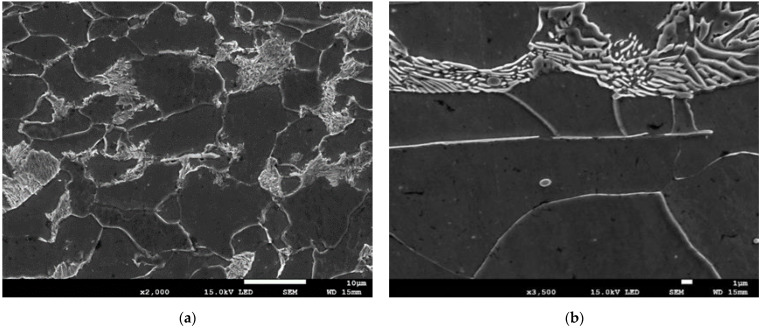
(**a**,**b**) Microstructure of S235 steel in the nondeformed condition.

The following figures show SEM images taken at different points of the elongated specimen. When the material of steel S235 had not been deformed (Figure 6), it consists of ferrite and pearlite grains with a size of 5–15 µm. The material includes elongated MnS inclusions, but no damage in these inclusions or decohesion between the inclusions and the ferritic steel matrix was observed.

**Figure 7:**
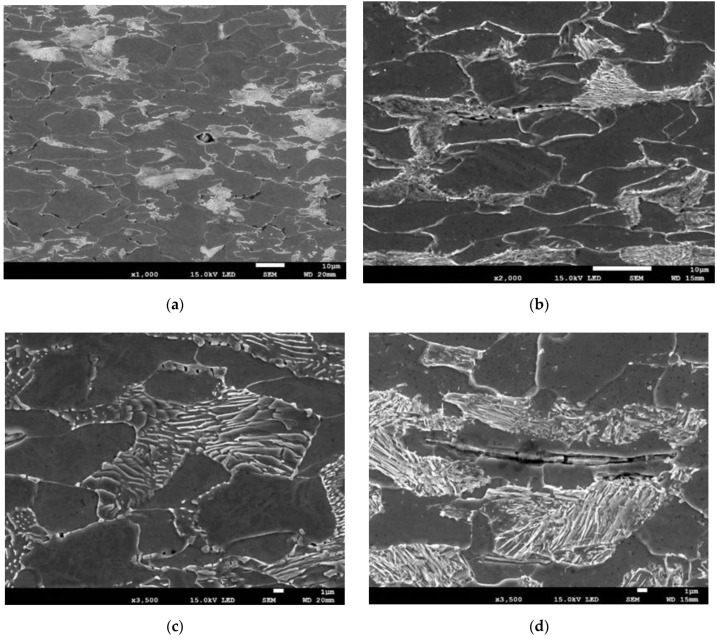
(**a**–**d**) Decohesion and fracture of MnS inclusions during the initial stage of neck formation.

At the moment corresponding to the beginning of neck formation, in addition to the elongation of ferrite grains, there was some decohesion between MnS inclusions and the ferritic matrix, as well as fracture of elongated MnS inclusions (Figure 7).

**Figure 8:**
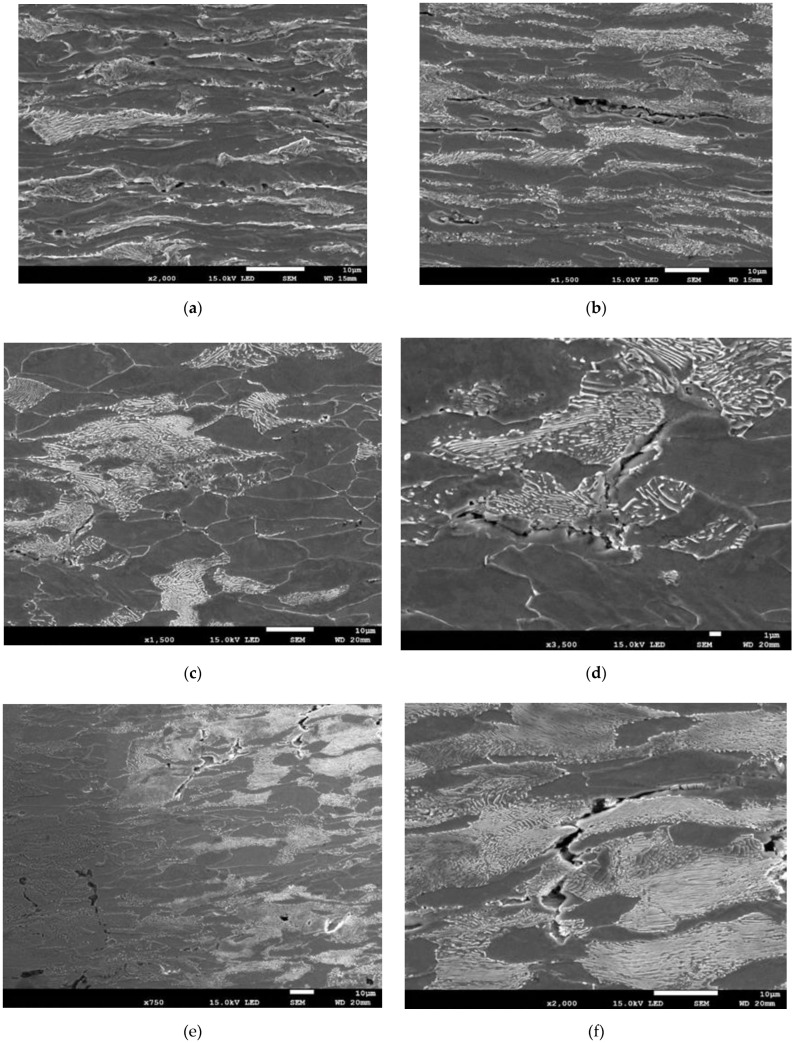
Nature of material damage in the specimen neck: (**a**–**d**) middle stage of neck formation; (**e**,**f**) final stage.

SEM images taken at different stages of neck formation in the specimen show how the damage developed in the material (Figure 8). During the stage of distinct neck formation (approx. half of the decrease in max. force value), numerous microcracks form in the material due to the connected microcracks formed at locations of non-metallic inclusions and at the pearlite/ferrite phase interface (Figure 8a–d). At this stage, there is a distinct dominance of laminar fractures between elongated ferrite grains. The process is clearly noticeable in the pictures of the sections exposing the plane across the pipe thickness (Figure 8a,b). In the photographs of sections of the plane normal to the pipe surface, the damage in the material is less visible (Figure 8c,d).

At the final stage, just before the tearing of the specimen, the microcracks merge, forming mesocracks perpendicular to the direction of tension. These mesocracks are formed through the tearing of elongated ferrite grains due to the shear mechanism, as clearly shown in the pictures of the plane with the normal orientation (Figure 8e,f).

**Figure 9:**
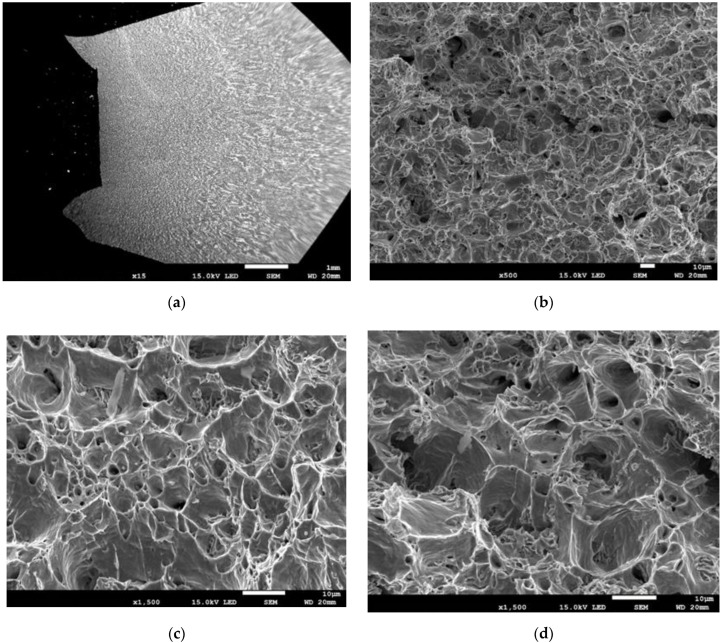
(**a**) Macro view of specimen microstructure nearest the fracture plane (picture across the thickness); (**b**–**d**) fracture relief of specimen breakthrough in the central part of the specimen (normal oriented pictures).

The mesocracks merge together with each other and with elongated voids, forming a macrocrack of the bottom of the fracture surface of the specimen (bottom of the cup), with the characteristic profile shown in photographs in Figure 9a–c. The final stage of specimen damage is the fracture of material areas next to the sidewalls of the specimen. After the formation of a macrocrack in the central part of the specimen section, the specimen adopts the form of a thin-wall tube whose walls are damaged due to shear at an angle of approx. 45° (Figure 9d).

### 3.3. Description of the Fracture Process

In the initial condition, the examined steel has a ferritic-pearlitic microstructure, with distinct banding of pearlite layers across the pipe wall thickness. There are also elongating inclusions of MnS flattened due to rolling and inclusions of oxides and nitrides in a spherical and regular shape (various polygonal types).

**Figure 10:**
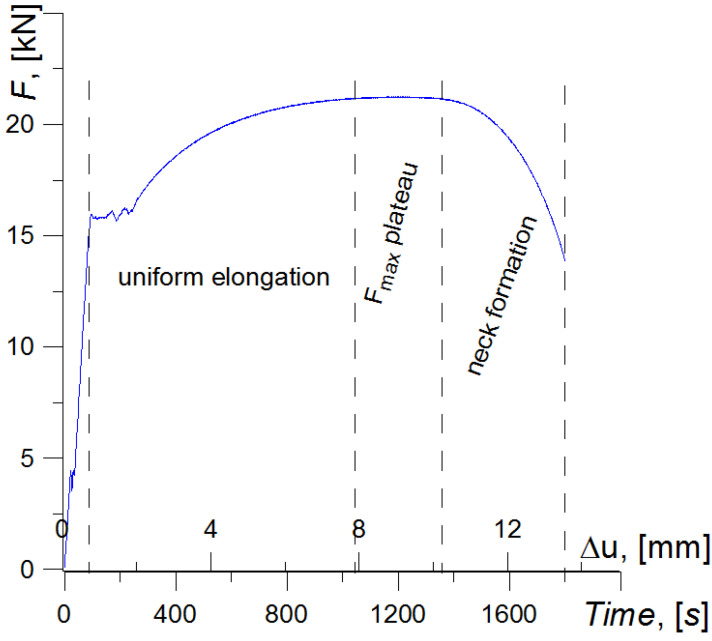
Schematic representation of the intervals of the material failure process during the uniaxial tensile test.

The chart of specimen loading during uniaxial elongation is shown in the figures (Figure 10). Various processes connected with strain and damage in the examined material, S235 steel, can be observed during loading at certain time intervals.

In the linear-elastic strain range, no permanent deformations or damage should appear in the material.

In the uniform strain range, material deformation occurs at the section between the yield stress or proof stress and the maximum force (time interval [100–1100 s]) due to the movement of dislocations.

The next range is the plateau on chart F—Δu, where the maximum load F_max_ is maintained at an almost constant level at time interval [1100–1350 s]. In this range, due to microstructural testing, a partial loss of coherence between the inclusions and the ferritic matrix of the material was observed, as well as cracking of the MnS inclusion particles and perlite platelets. The stabilization of the load level indicates the location of the deformation process on a limited section of the specimen.

The reduction in the force loading of the specimen marks the beginning of neck formation (time interval [1350–1800 s]) (Figure 10). At this stage, the deformation process is located in a small section, the size of which decreases during neck development. Due to the reduction in the active section, the strain processes become more local and occur in an increasingly smaller volume of the material. In this area, the level of true stresses and strains radically increases [34]. This intensifies the delamination and fracture of inclusions and pearlite plates, even including damage between the grains. In the remaining areas of the specimen material, in turn, the load is reduced, and there are no further processes connected with strains and damage development.

The last short stage is the fracture of the specimen, which was realized in time interval [1800–1810 s].

### 3.4. Acoustic Emission (AE) Signals Analysis

**Figure 11:**
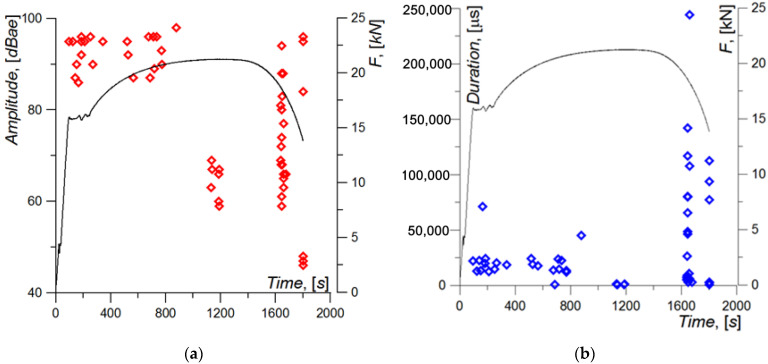
Characteristics of the AE signal recorded during specimen loading: (**a**) Amplitude; (**b**) Duration.

**Figure 12:**
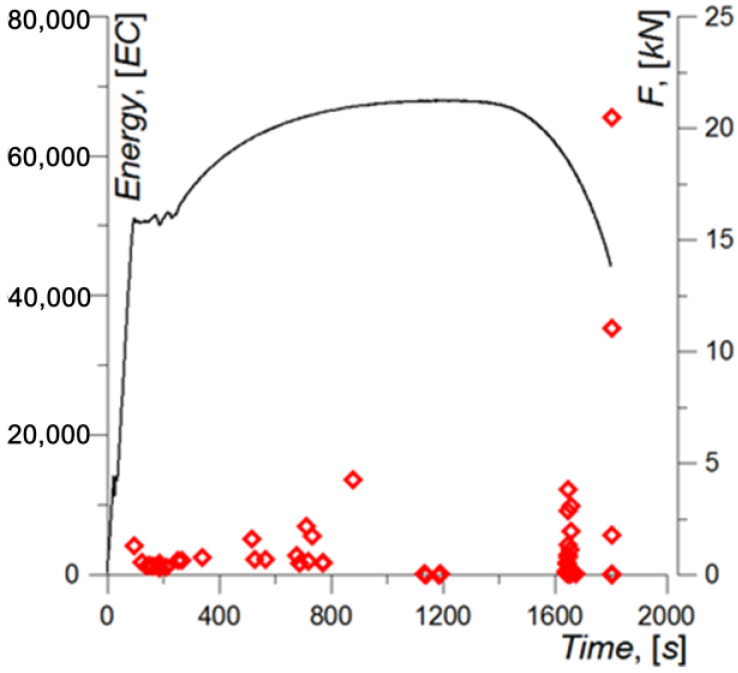
Energy AE parameter recorded during specimen loading.

**Figure 13:**
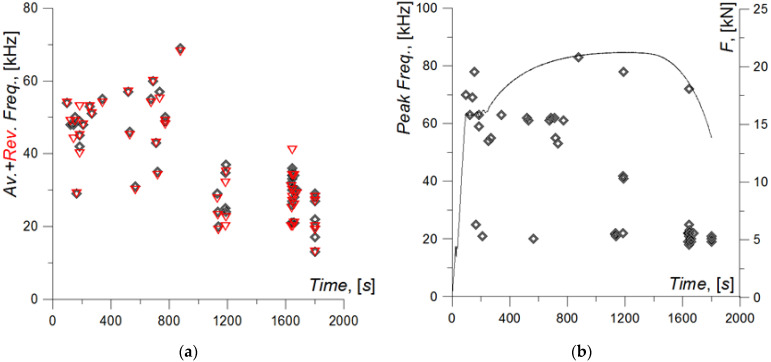
Frequency characteristics of the AE signal recorded during specimen loading: (**a**) Average (black symbol) and Rev. Freq. (red symbol); (**b**) Peak Freq.

AE signals were present during the uniaxial elongation of the specimen virtually throughout the loading range (Figure 11, Figure 12 and Figure 13). The figures below show the basic characteristics of AE signals during sample loading. Material damage processes were analyzed based on signals with amplitude >40 dB (Figure 11a).

The amplitude characteristic of the AE signal shows that sounds with the highest level occur in the initial phase when plastic strain occurs along the entire length of the tested section of the specimen [t < 1000 s] and at the final stage of the neck formation and during specimen failure [t > 1600 s]. In the middle phase, in turn, directly before neck formation in the specimen [t: 1000–1600 s], the amplitude characteristic of the AE signals decreases (Figure 11a).

The distribution of the duration characteristic is slightly different (Figure 11b). In the initial loading stage, the values are approximately within the range of 12,000–25,000 µs, and some of them reach approx. 50,000 µs and approx. 75,000 µs. In the middle loading phase, the duration characteristic is reduced significantly to approx. 500–1300 µs. During the stage of distinct neck formation, there are signals with very high values—more than 100,000 µs or even 244,440 µs. At the time of failure, the max. values of the duration characteristic reach [75,000–112,000 µs].

The highest levels of the energy parameter (Figure 12) were recorded upon specimen failure, and the lowest—in the time interval corresponding to the beginning of neck formation.

Figure 13 shows the frequency characteristics of AE signals: average and reverberation frequency and peak frequency. The curves indicate that the aver. and rev. freq. characteristics are very similar. This indicates that the average frequencies and reverberation frequencies are similar, which means that the recorded events are stabilized signals. The data included in Figure 13a clearly show that the analyzed frequency characteristics of AE signals start to decrease from the time of neck formation in the specimen. During neck formation, the characteristics are within the range of approx. 15–40 kHz. In the range of uniform plastic elongation, in turn, they are within the range of approx. 30–70 kHz.

Since the dominant phenomena occurring in the specimen material during loading until the ultimate strength of the specimen is reached (until F_max_, until 1100 s) are plastic strains, we can assume that AE signals with frequency characteristics aver. and rev. freq. over 30 kHz are connected primarily with the processes related to plastic strains, i.e., the movement of dislocations. In the AE signals recorded in the second section of the load curve, when neck formation and specimen failure occur, there is a dominance of frequencies lower than 30 kHz for the aver. and rev. freq. characteristics. In that interval, there are also signals caused by plastic strains—during ductile fracture, the material deforms plastically, and AE signals with aver. and rev. freq. higher than 30 kHz may be emitted by plastic strain processes in the material.

The peak freq. characteristic, which corresponds to the frequency at the time of the peak with the highest amplitude, is distinctly split into two levels: approx. 15–25 kHz and approx. 50–80 kHz, which can be observed throughout the specimen strain process (Figure 13b). However, in the range of uniform elongation, there is a dominance of signals with peak freq. of approx. 50–80 kHz, and during the stage of neck formation and specimen failure—approx. 15–25 kHz.

**Figure 14:**
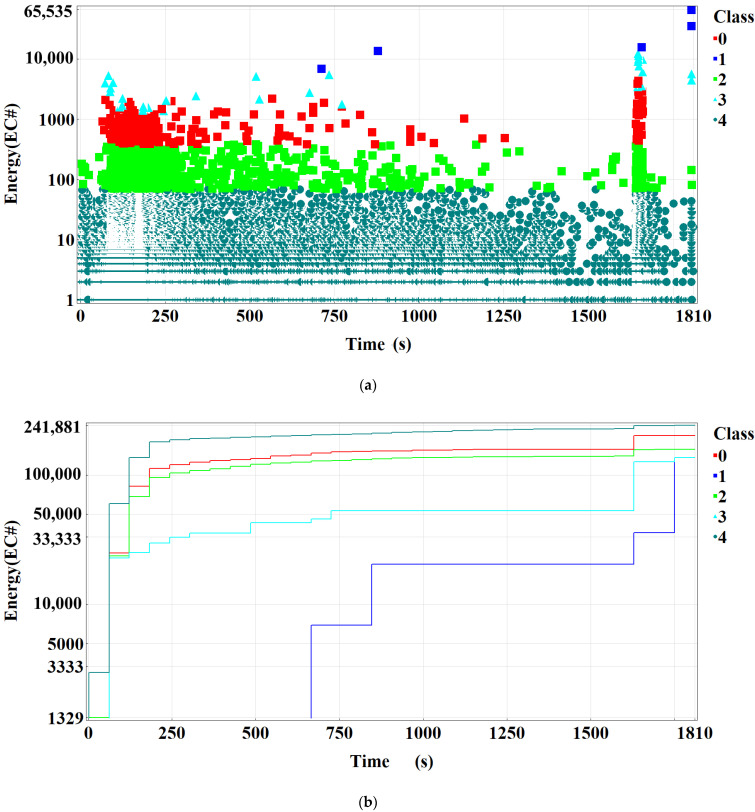
Chart of acoustic emission signal classes over time for the energy parameter: (**a**) point chart, (**b**) cumulative chart.

Figure 14 shows the graphical results of the analysis carried out based on the *k-means* clustering method [16,17,18,19], and Table 2 shows the range of respective characteristics of AE signals. The selected clustering method enabled a breakdown of AE signals recorded during the measurements into a pre-selected number of classes and association of the classes with processes occurring in the material structure, as discussed in Refs [16,17,18,19,20,21,22,23,24]. The measure of the fit of signals to the individual classes was the R^2^ coefficient, which amounted to no less than 0.9 in each case [25,26,27,28,29,30,31,32,33].

The use of the *k-means* algorithm to divide the acoustic emission signals into clusters enables their quick processing, due to the size of the measurement files and the number of parameters used for the analysis (14) [32].

In Figure 14a, the recorded signals are shown as discrete points in the energy–time coordinate system. Figure 13b presents the cumulative curves of energy in time. This type of graph shows well the increase in analyzed parameter. The highest values correspond to signals of class 1 (Table 2). Signals of class 1, despite their low number, have the highest energy and strength. Relatively high values of AE signal parameters were also recorded for signals of class 3 and class 0. The signals with lower values of the parameters are included in class 2 and class 4.

Class 4, which is the largest class, groups low-energy signals (Table 2, Figure 14b). The biggest number of signals of this class was recorded at the initial loading stage—still in the linear-elastic strain range. During the next stage, their number decreases, and the signals disappear during neck formation. The nature of the change of these signals may suggest that they are connected with the loading arrangement (during the quick increase in the load, more signals are generated), which means that they may be caused by the friction between the rollers and the specimen.

Signals of classes 0 and 2 have a very similar distribution during the loading of the specimen (Figure 14b). They differ in the max. value of energy parameters, but they have a similar frequency range (Table 2). These signals may characterize the same process. The form of signals of class 3 is also very similar to classes 0 and 2—however, in their case, there is a smaller (more compact) frequency range and higher levels of AE signal strength. The coverage and distribution of class 0, 2, and 3 signals are similar. It was assumed that they are emitted by successive processes occurring when loading the sample—plasticization, the formation of local microcracks and the development of microcracks.

Signals of class 4 were present throughout the entire specimen loading range, and signals of classes 0, 2 and 3 were recorded primarily during the uniform elongation (strain) of the specimen and at one point during neck formation.

AE signals of class 1 are different from the other signals. The signals of this class were only recorded before the beginning of neck formation, during its formation and at the time of specimen fracture. During loading, the number of recorded signals of this class was low, but they had high energy and strength characteristics, particularly before breaking and at the time of specimen fracture.

The results shown in Figure 13 clearly show that the increase in strain in the specimen material is associated with a tendency for reduced frequency characteristics of AE signals, i.e., in the process of neck formation and specimen fracture. The detailed frequency analysis of AE signals was conducted using methods based on time-frequency analysis and wavelet analysis.

Wavelet transformation (wavelet analysis), which is a type of time-frequency analysis, thanks to the possibility of analyzing changes in signal frequency over time, is an advanced tool for the processing and analysis of AE signals, particularly non-stationary and transient signals. The wavelet transform does not have any of the flaws of the commonly known and used Fourier transform. That is because this method does not result in the loss of information about time but enables the simultaneous presentation of time and frequency dependencies between the components of the analyzed signals. The wavelet transform also approximates the signals by identifying their characteristic structural elements.

**Figure 15:**
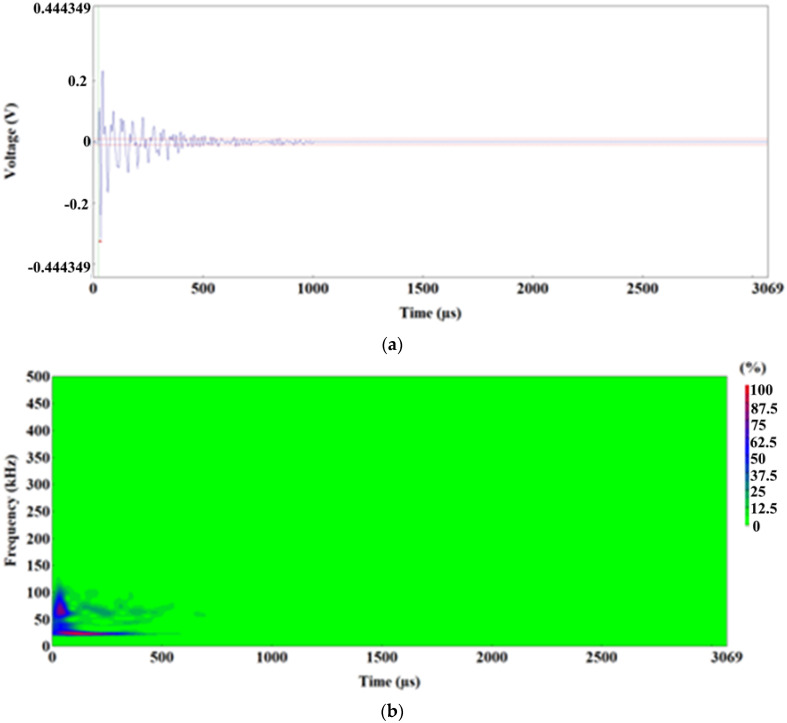

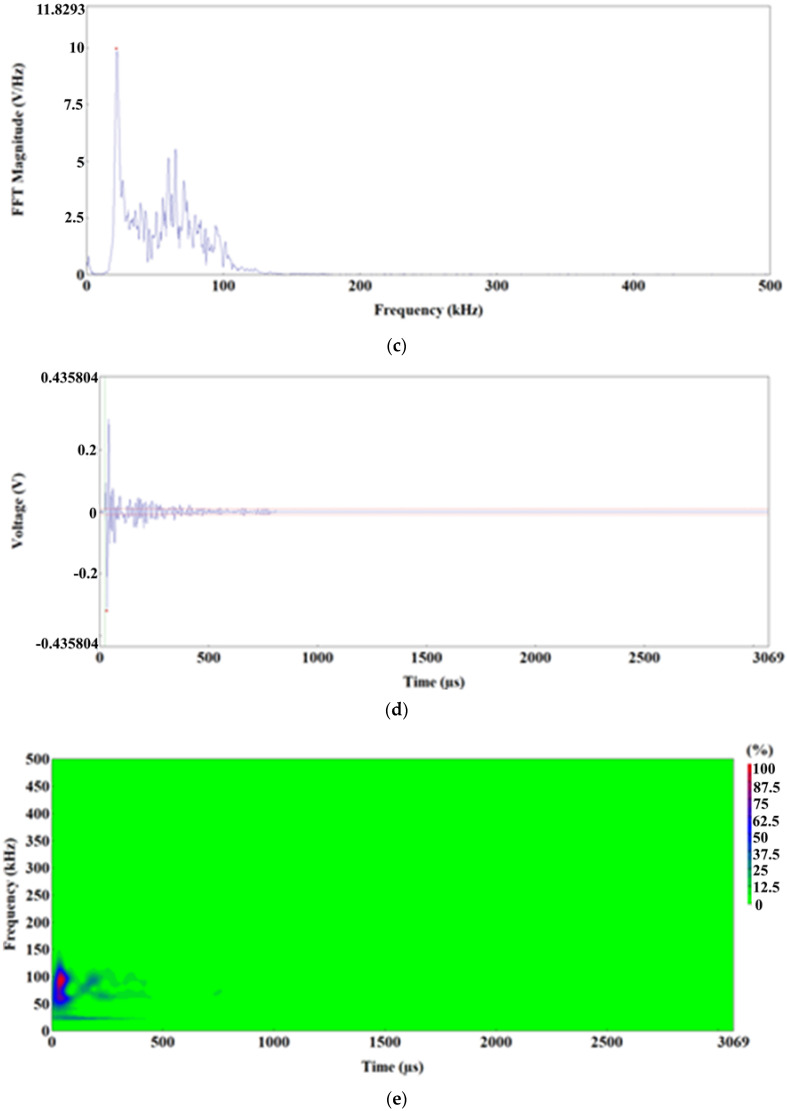

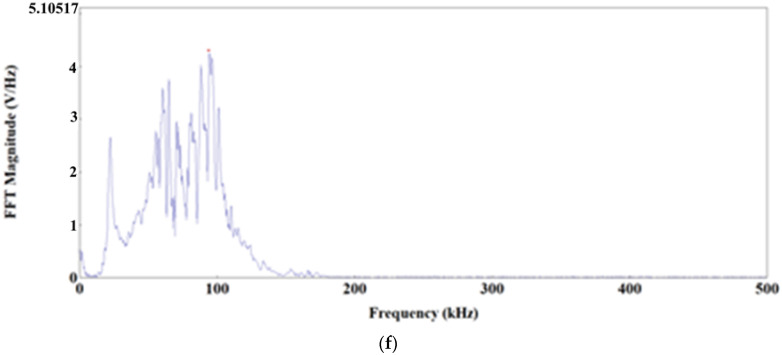
Time-frequency spectra for the 1100–1200 s time range: (**a**,**d**) Waveform Time Domain graphs, (**b**,**e**)Waveform Continuous Wavelet graphs, (**c**,**f**) FFT Magnitude graphs.

**Figure 16:**
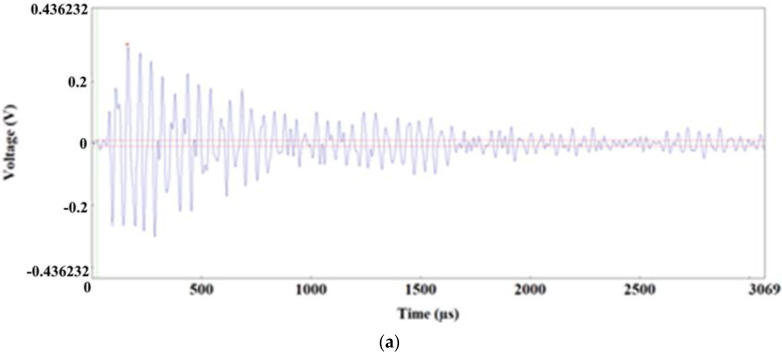

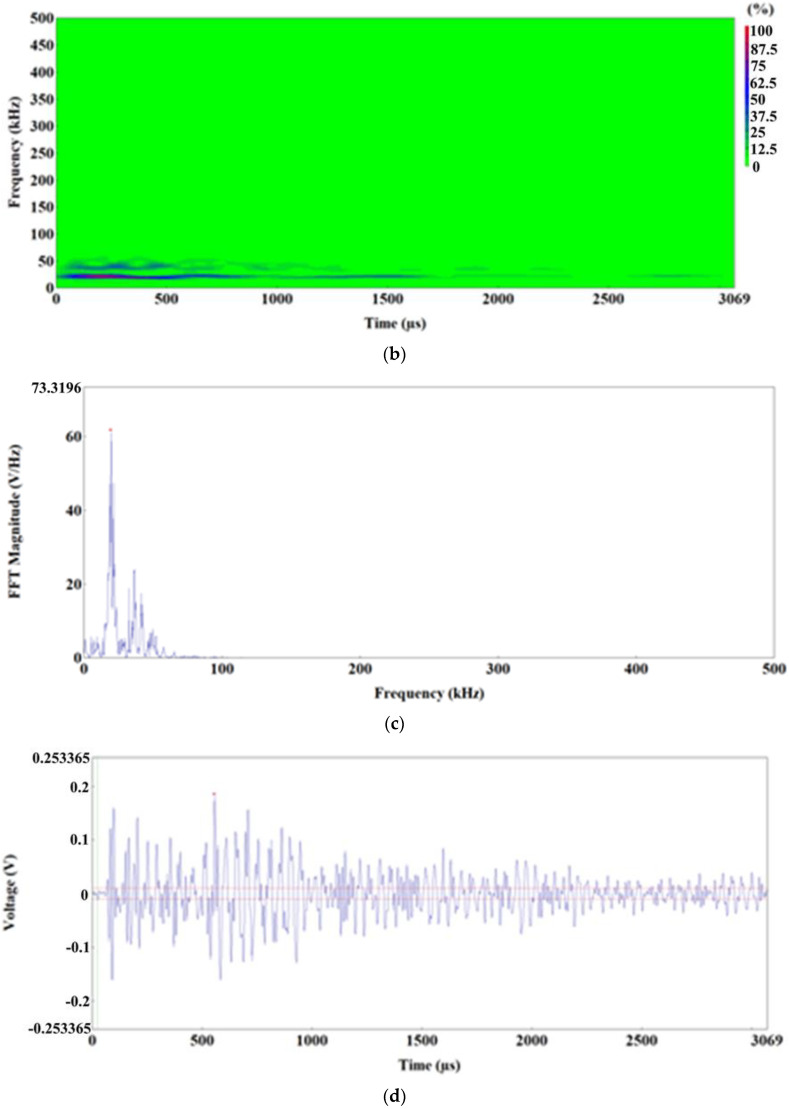

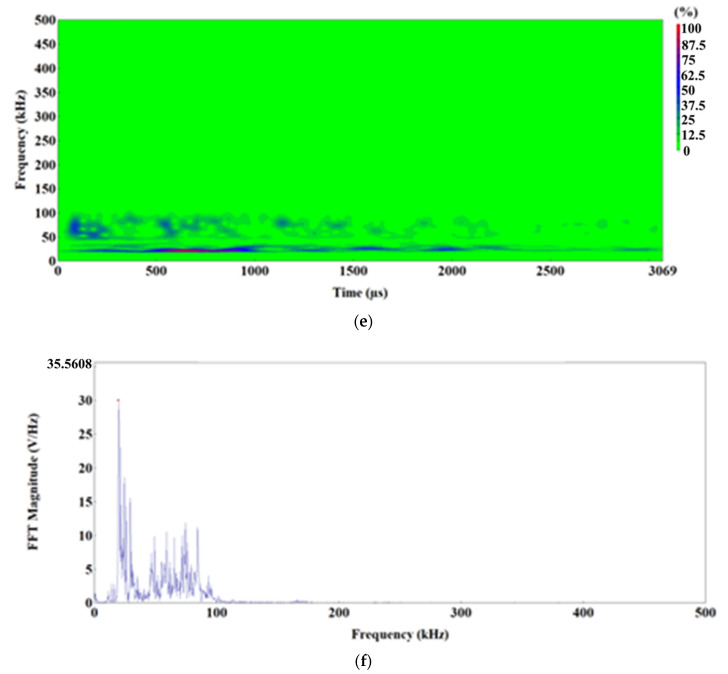
Time-frequency charts for the 1600–1700 s time range (distinct neck formation in the specimen): (**a**,**d**) Waveform Time Domain graphs, (**b**,**e**)Waveform Continuous Wavelet graphs, (**c**,**f**) FFT Magnitude graphs.

**Figure 17:**
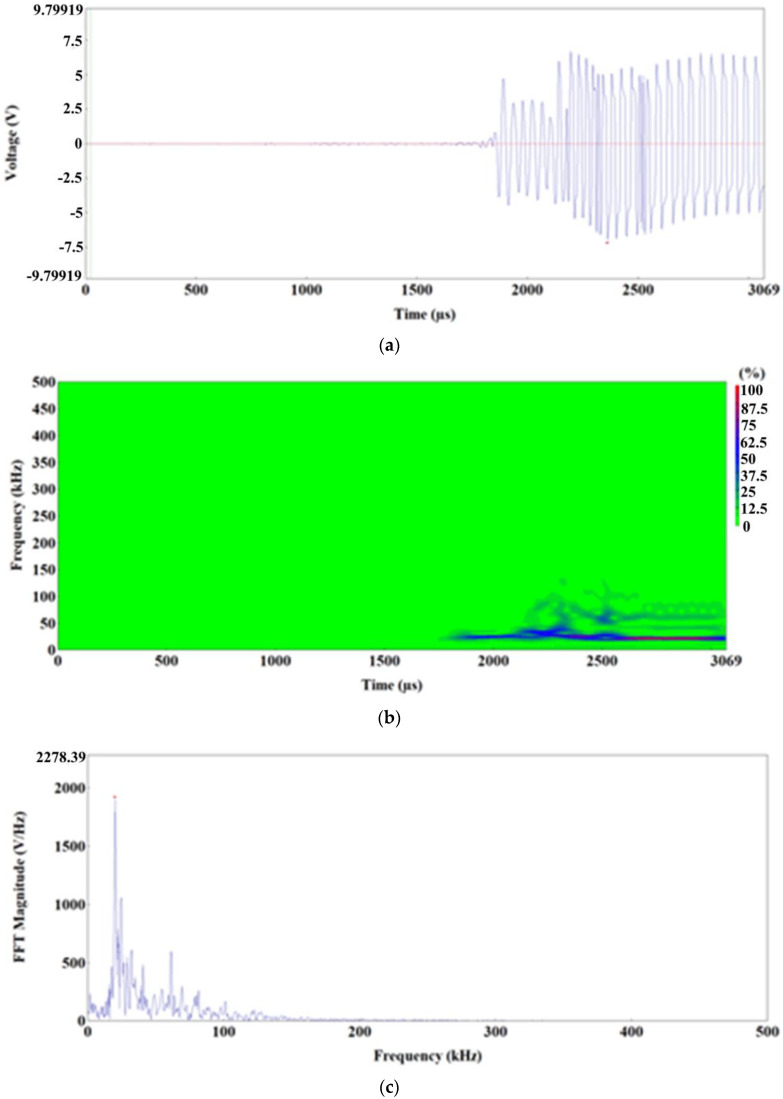

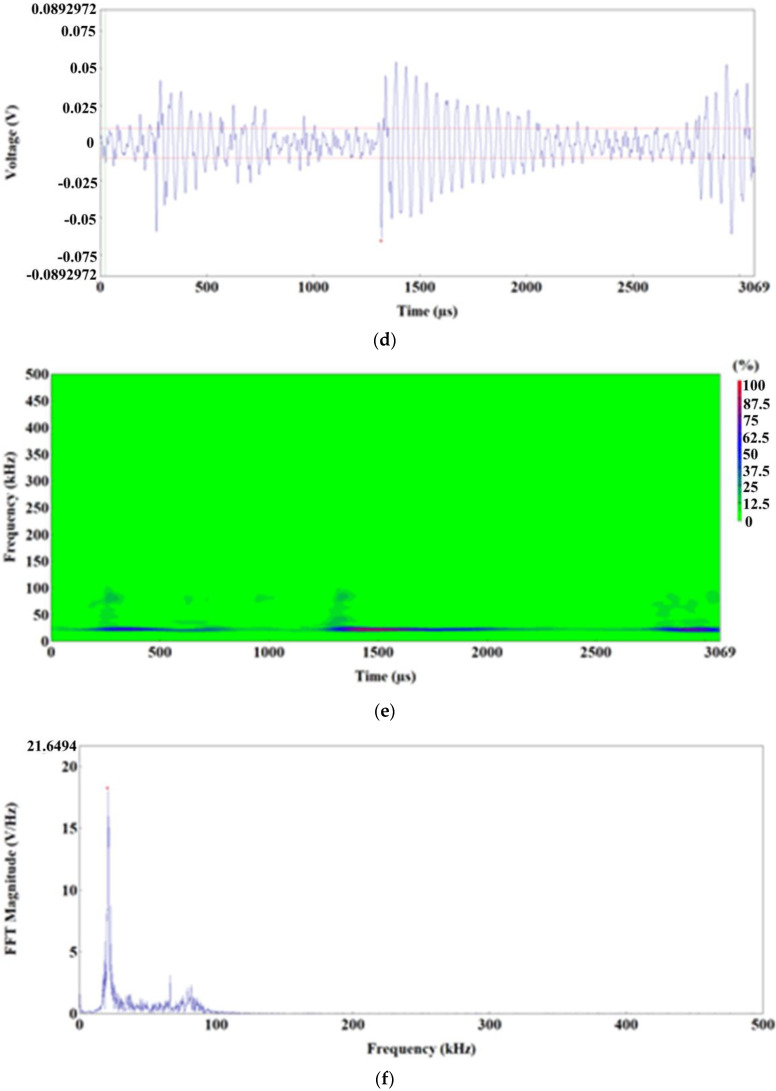
Time-frequency charts for the time of specimen failure: (**a**,**d**) Waveform Time Domain graphs, (**b**,**e**)Waveform Continuous Wavelet graphs, (**c**,**f**) FFT Magnitude graphs.

Figure 15, Figure 16 and Figure 17 and Table 3 show the forms of selected AE signal characteristics.

In the uniform elongation range, the dominant phenomenon occurring in the material were plastic strains. Damage in the material is insignificant; it only manifests in individual acts of decohesion between an inclusion and the ferritic matrix and (or) fracture of large elongated MnS inclusions. This is accordingly reflected by the characteristics of AE signals.

Most of the signals recorded in this range have the characteristic shape shown in Figure 15a. The dominant frequency range of these signals is 40–80 kHz, with the presence of magnitude peaks at 50–60 kHz (Figure 15b,c); the duration of these signals is 12,000–25,000 µs; energy is = 1000–7000 EC.

At the final stage of the range of uniform elongation of the specimen, when almost the maximum load levels were reached (immediately before the commencement of neck formation in the specimen), AE signals with a slightly different character were observed. A sample form was shown in Figure 15d–f. For these signals, there are values in the ranges of approx. 25–35 kHz and 50–100 kHz in the frequency spectrum, but the dominant frequencies are in the range of 60–100 kHz. These signals are characterized by higher values of the following parameters: duration = 45,263 µs; energy = 13,516 EC.

The next cluster of AE signals corresponds to the stage of distinct neck formation in the specimen (1600–1700 s). The characteristic shapes and frequency spectra are shown in Figure 16a–f. Generally, signals from this range have low voltage (up to 0.6 V) and long duration (50,000–245,000 µs). The frequency spectrum indicates that magnitude peaks occur in ranges of 25–30 and 50–90 kHz, and maximum signal magnitudes are achieved in the 25–30 kHz frequency range.

Figure 17a–f shows the AE signals and their frequency spectra at the time of specimen failure. All signals are located in a time interval smaller than 0.5 s, which indicates that the failure process occurs suddenly. The AE signals from that range have a long duration (up to 113,000 µs) and reach the highest recorded energy, up to 65,500 EC. The maps and charts of frequency spectra indicate that the highest magnitudes of AE signals were recorded in the 18–30 kHz frequency spectrum. At higher frequencies, the magnitude of the signal was lower.

## 4. Conclusions

The above results of tests of the fracture process in ferritic-pearlitic S235 steel carried out through the observation of microstructural changes (metallographic examinations) and analysis of AE signals support a few general conclusions.

- Various material damage mechanisms can be observed during the uniaxial tensile loading of the specimen: plastic strain in the matrix (primarily in ferrite grains), decohesion between the inclusions and the ferritic matrix of the material, fracture of MnS inclusions and coagulated precipitates at grain interfaces and fracture of ferrite grains.
- Plastic strain is present at every stage of damage evolution in the material (S235 steel) during specimen elongation. It is also the dominant process at the stage of uniform elongation in the specimen until the maximum strength is reached. The band of dominant frequencies of typical AE signals in that range corresponds to 50–100 kHz. Sporadically, lower frequencies were also observed in the spectra of the signals, but the magnitudes for the lower frequencies were also significantly lower. Consequently, it was found that signals with a frequency of 50–100 kHz characterized plastic strain in the material. Plastic strain at this stage was accompanied by local fractures or decohesion, but these phenomena occurred sporadically, and their intensity was low.
- In the range of maximum specimen strength (beginning of neck formation), the frequency spectra of AE signals also include 50–100 kHz frequencies. The same spectra also had a significant content of 18–36 kHz frequencies. In the case of some signals, there was a dominance of the 50–100 kHz frequency range, and in others—18–36 kHz. Therefore, it was found that the recorded signals were generated by two mechanisms: plastic strain and, most likely, decohesion and fracture of some of the largest MnS inclusions. The fracture of these inclusions reduces the effective cross-section of the specimen, increasing stresses and strains in the local area. The increase in stresses and strains in this local area, in turn, causes the fracture of small precipitates.
- At the next stage of loading (when the neck is already well formed and visible), the fracture mechanism of MnS inclusions intensifies significantly. The spectra become dominated by the 18–36 kHz frequency range. Frequencies in the range of 50–100 kHz are also present to a small extent, which indicates the development of plastic strains in local areas. It was found that AE signals recorded at this stage were primarily caused by the fracture of MnS inclusions and decohesion between MnS inclusions and precipitates at the grain interface. These mechanisms generate frequencies in the range of 18–36 kHz. They are accompanied by plastic strains, which generate signals with a frequency of 50–100 kHz.
- AE signals in the range of 18–35 kHz were recorded at the last stage, i.e., specimen failure (fracture), and they made up a significant portion of the frequency spectrum. This spectrum also included the highest signal voltage magnitudes. Frequencies of 50–100 kHz are also present in the spectra to a small extent, indicating a small contribution of the signals emitted by the plastic strains in the material to the fracture mechanism.
- Class 1 acoustic emission signals can be related to the onset of neck formation before component failure.
- Correlation was found between the recorded frequency spectra and the mechanical parameters of the steel material.
- The analysis of the acoustic emission signal classes and the frequency of the event spectra enables the assessment of the steel condition and the detection of a potential hazard to an element or structure at an early stage.

As a result of the comprehensive application of different research methods—experimental tests, metallographic examinations of the microstructure and the analysis of AE signals, it was possible to determine the characteristics and frequency ranges of AE signals corresponding to the mechanisms of damage evolution in S235 steel used to make gas pipelines.

Detailed metallographic examinations of the most strained areas of the specimens made it possible to determine the type and extent of material damage, whereas the time-frequency analysis of AE signals using the *k-means* clustering method for the signals recorded during specimen loading made it possible to determine the frequency ranges of signals corresponding to different steel damage mechanisms.

The approach to the analysis of the development process of the destruction of the material of a structural element (gas pipeline in the current test) presented in this article allows, on the basis of the recorded AE signals, for indicating not only the moment of destruction but mainly assess the level of material destruction at which the tested object is. The occurrence of signals characteristic of the uniform elongation interval will mean that plasticization takes place in the material of the element, which is already a matter of concern for the user. The presence of signals characteristic of the next interval, plateau F_max_, means that there is local limited destruction of material—so the element is at risk. The presence of signals characteristic of the neck-forming interval means a high degree of damage risk—the element should be taken out of use immediately.

## Figures and Tables

**Table 1:** Mechanical characteristic of S235 steel.

Specimens	σ_YS_L_ [MPa]	σ_YS_H_ [MPa]	σ_UTS_ [MPa]	E [GPa]	A5 [%]	Z [%]
S235_01	386.20	393.12	510.62	191	25.77	62.74
S235_02	383.46	405.86	509.90	190	26.27	63.37
S235_03	384.04	395.00	520.71	199	27.30	63.87

σ_YS_L_—low value of yield strength_;_ σ_YS_H_—high value of yield strength; σ_UTS_—ultimate value of strength; E—Young modulus; A5—relative elongation of specimen; Z—relative reduction in specimen cross-section area.

**Table 2:** Ranges of the change of AE signal characteristics for respective clustering classes.

Class	Signal Strength (pV∙s)	Max. Amplitude (V)	Max FFT Real (V)	Frequency (kHz)	Max Energy (eu)	Max. Duration (µs)
**0**	2.41 × 10^6^–2.68 × 10^7^	96	180	20–76	4297	819
**1**	4.31 × 10^7^–9.58 × 10^8^	98	2000	22–69	65,535	32,523
**2**	4.36 × 10^5^–2.42 × 10^6^	90	20	16–63	388	162
**3**	8.61 × 10^6^–7.64 × 10^7^	96	250	17–61	12,237	3114
**4**	0–4.38 × 10^5^	78	8	2–3000	70	31

**Table 3:** Characteristics of the parameters and frequency ranges of AE signals at respective stages of specimen loading.

Loading Interval Time [s]	Uniform Elongation	Fmax Plateau	Neck Formation	Destruction
	[100–900]	[1100–1200]	[1600–1700]	[1802, 1803]
ASL	50–68	30–50	35–63	81
Amplitude	89–98	59–69	69–80; 88; 94	46–48; 95; 96; 98
Duration [μs]	12,000–45,260	500–1277	50,000–244,447	900–2900; 112,666; 93,884
Energy	1000–13,500	7–30	240–12,237	14–35; 35,246; 65,535
Signal Strength [pV∙s]	5.77 × 10^6^–8.44 × 10^7^	4.57 × 10^4^–2.32 × 10^5^	6.27 × 10^5^–7.64 × 10^7^	1.0 × 10^5^–2.25 × 10^5^; 2.2 × 10^8^; 9.58 × 10^8^
Frequency [kHz]	25–35; 50–80	25–35; 50–100	25–30; 50–90	18–30; 50–90
Frequency for Magnitude Peaks [kHz]	50–65; 60–100	25–35; 50–100	25–30;	18–30

## Data Availability

Not applicable.
